# Oral probiotics increased the proportion of Treg, Tfr, and Breg cells to inhibit the inflammatory response and impede gestational diabetes mellitus

**DOI:** 10.1186/s10020-023-00716-4

**Published:** 2023-09-08

**Authors:** Weijie Liang, Yuanyi Feng, Dongmei Yang, Jiajun Qin, Ximei Zhi, Wen Wu, Qiang Jie

**Affiliations:** 1 The Second School of Clinical Medicine, Southern Medical University, Guangzhou, 510515 People’s Republic of China; 2 Department of Geriatric Endocrinology, Guangdong Provincial Geriatrics Institute (East Zone), Guangdong Academy of Medical Sciences, Guangdong Provincial People’s Hospital, No. 3, Chanchugang, Zhongshan 2nd Road, Yuexiu District, Guangzhou, 510080 Guangdong Province People’s Republic of China; 3 Department of Cardiology, Panyu Central Hospital, Cardiovascular Institute of Panyu District, No. 8, Fuyu East Road, Qiaonan Street, Panyu District, Guangzhou, 511400 Guangdong Province People’s Republic of China; 4 Department of Geriatrics, Huazhong University of Science and Technology Union Shenzhen Hospital (Nanshan Hospital), Shenzhen, 518052 People’s Republic of China

**Keywords:** Gestational diabetes mellitus, Gut microbiota dysbiosis, Leptin, Probiotics, Transcriptome sequencing, Metagenomic sequencing, Inflammatory response

## Abstract

**Background:**

Children of mothers with gestational diabetes mellitus (GDM) are more prone to acquire type 2 diabetes and obesity as adults. Due to this link, early intervention strategies that alter the gut microbiome may benefit the mother and kid long-term. This work uses metagenomic and transcriptome sequencing to investigate how probiotics affect gut microbiota dysbiosis and inflammation in GDM.

**Methods:**

GDM and control metagenomic sequencing data were obtained from the SRA database. This metagenomic data helped us understand gut microbiota abundance and function. KEGG detected and extracted functional pathway genes. Transcriptome sequencing data evaluated GDM-related gene expression. Finally, GDM animal models were given probiotics orally to evaluate inflammatory response, regulatory immune cell fractions, and leptin protein levels.

**Results:**

GDM patients had more Fusobacteria and Firmicutes, while healthy people had more Bacteroidetes. Gut microbiota composition may affect GDM by altering the L-aspartate and L-asparagine super pathways. Mannan degradation and the super pathway of L-aspartate and L-asparagine synthesis enhanced in GDM mice with leptin protein overexpression. Oral probiotics prevent GDM by lowering leptin. Oral probiotics increased Treg, Tfr, and Breg cells, which decreased TNF-α and IL-6 and increased TGF-β and IL-10, preventing inflammation and preserving mouse pregnancy.

**Conclusion:**

Dysbiosis of the gut microbiota may increase leptin expression and cause GDM. Oral probiotics enhance Treg, Tfr, and Breg cells, which limit the inflammatory response and assist mice in sustaining normal pregnancy. Thus, oral probiotics may prevent GDM, enabling targeted gut microbiota modulation and maternal and fetal health.

**Supplementary Information:**

The online version contains supplementary material available at 10.1186/s10020-023-00716-4.

## Introduction

Gestational diabetes mellitus (GDM) is a common pregnancy complication associated with poor maternal and fetal outcomes (Ye et al. 2022). It is one of the prevalent metabolic complications during pregnancy and is associated with an elevated risk of adverse pregnancy outcomes in the mother and her offspring (Zietek et al. 2021). GDM is characterized by impaired glucose tolerance caused by maternal pancreatic beta-cell dysfunction, resulting in inadequate regulation of glucose homeostasis by insulin during pregnancy (Deischinger et al. 2020).GDM is associated with low-grade inflammation and intestinal microbiota (Mustad et al. 2020). The changes in the gut microbiota play a decisive role in the development of obesity, insulin resistance, and chronic inflammation (Ionescu et al. 2022). The gut microbiota has been shown to contribute to all aspects of host physiology, from immune regulation to drug metabolism, and alterations in gut microbiota composition are responsible for many diseases as well as responses to drugs (Schilcher and Horswill 2020; Yang et al. 2020; Wang et al. 2022a, b, c, d, e). During pregnancy, GDM patients experience dysbiosis of the gut microbiota and a decrease in the number and impaired function of regulatory immune cells such as Treg cells. This disruption causes abnormal inflammatory responses in pregnant women and leads to various prenatal complications in the fetus (Schober et al. 2014; Paolino et al. 2021). Probiotics are valued for influencing the composition of the intestinal microbiota and improving the integrity of the gut (Wieers et al. 2019). Probiotics are a promising tool to reduce the frequency of GDM in pregnant women by enhancing the balance of the intestinal microbiota and inhibiting the expression of inflammatory factors (Wieers et al. 2019). However, their understanding of their action mechanism is still very limited (Wang et al. 2022a, b, c, d, e). Therefore, mechanistic studies are needed to identify targets for GDM prevention through probiotic microbial modulation of the gut microbiota (Feng and Liu 2022).

Notably, dysbiosis of the gut microbiota and their metabolites may trigger insulin resistance in diabetes by driving the inflammatory response (Dabke et al. 2019). The gut microbiota is the gastrointestinal tract, specifically the colon, with the highest microbiota density (Lee et al. 2022). Gut microbiota dysbiosis is an imbalance between commensals and pathogens associated with various diseases, including GDM (Zuo et al. 2020). Previous evidence documents significant differences in gut microbiota composition between patients with metabolic disorders, such as obesity and type 2 diabetes mellitus (T2DM), and healthy individuals (Sharma et al. 2021). Another study also showed that the abundance of gut microbiota in GDM patients was abnormal at both the phylum and genus levels compared to healthy controls (Chen et al. 2021a, b). In addition, probiotics play an important role in regulating the gut microbiota composition in patients with GDM (Ding et al. 2021).

In addition, advances in metagenomics have enabled detailed studies of the role of the gut microbiome in human health and disease, including GDM (Bai et al. 2021). As a result, metagenomic sequencing data were obtained to allow the following analysis of the composition, abundance, and functional makeup of the gut microbiota in GDM patients and healthy individuals (Liu et al. 2021). In addition, previous studies have shown that leptin, which is highly expressed in the placenta, is involved in the development of GDM (Sweeting et al. 2022). There is evidence that gut microbiota dysbiosis is associated with leptin expression under inflammatory conditions, but the exact interactions remain understood (Rodriguez-Mejia et al. 2022).

**Fig. 1:**
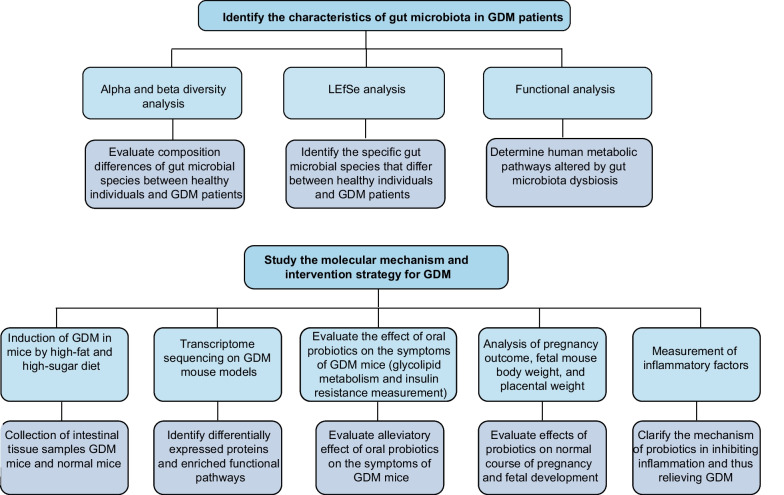
A flow chart of the study procedures

This study examined differences in gut microbiota composition, species abundance, and functional composition between GDM patients and healthy individuals and found significant differences (Fig. 1). It also investigated the possible mechanisms behind the development of GDM due to gut flora dysbiosis. The study further demonstrated the therapeutic potential of oral probiotics to prevent GDM by modulating leptin levels and modulating the immune response to suppress inflammation and maintain normal pregnancy. These findings provide a basis for further research into the pathophysiology of GDM and the development of novel interventions for clinical management and prevention.

## Materials and methods

### Sequence read archive (SRA) database GDM-related metagenomic sequencing

Fecal samples (PRJNA401977) from 75 GDM patients and 70 healthy individuals were obtained through the SRA database (https://www.ncbi.nlm.nih.gov/sra/) in NCBI, from which 5 cases of GDM patients and healthy individuals were randomly selected for metagenomic analysis. The sample size selected for this study was determined based on the principles of metagenomic sequencing and previous research (Forster et al. 2019).

### Analysis of the abundance of the microorganisms

To remove host and contaminated sequences, samples were assessed using multiQC for sequence quality control and kneaddata (https://github.com/biobakery/biobakery/wiki/kneaddata). The microorganism tree was drawn by GraPhlAn (https://github.com/biobakery/graphlan.git) to obtain the relative abundance of microbial classification, followed by Alpha diversity analysis using richness and Shannon index. The Wilcoxon rank-sum and Welch t-test were used to compare bacterial abundance and diversity. Bar graphs of all differential abundances were plotted by LEfSe (http://huttenhower.sph.harvard.edu/lefse/) analysis, with a linear divergence analysis (LDA) score threshold 2.0. The LDA scores show the degree of influence of the species that significantly differ between the different groups, with higher scores indicating greater differences in characteristics between the two groups.

### Analysis of the microbial functional composition

The pathway abundance table, including functional pathways and species composition, was obtained through HUMAnN2 (https://github.com/biobakery/biobakery/wiki/humann2), which indicated that the stratified (species relative abundance of unclassified) and unstratified (species relative abundance of classified) results were acquired. The statistical analysis and visualization were performed using STAMP software (version: v2.1.3; https://beikolab.cs.dal.ca/software/STAMP). The Welch t-test was used to compare functional composition differences.

### Bacterial strain preparation

Bacillus strains FTJS7K1 and FTJS5K1 were isolated from stool samples of healthy individuals. They were cultured and purified for 48 h at 37 °C, under anaerobic conditions, on improved brain-heart infusion (BHI) agar plates supplemented with 2% agar and pH adjusted to 7. After incubation, a single clone was selected and cultured for 18 h in a BHI medium (pH 7, 37 °C). Subsequently, the bacterial suspension was centrifuged at 6000 rpm for 10 min to collect the strains, which were then washed three times with 1×PBS and finally resuspended in 1×PBS to obtain a final concentration of 1 × 10^9^ colony-forming units (CFU). The healthy individuals included in the study were voluntary participants of our hospital’s health examination center, with 10 individuals (5 males, 5 females), with an average age of 25 years. In total, 10 stool samples were collected from these healthy individuals.

### Induction of GDM mouse models

C57BL/6J mice (90 female mice and 30 male mice; aged 8 weeks old; weighing 20–25 g) were purchased from Vital River Laboratory Animal Technology Co., Ltd. (Beijing, China). Mice were fed with common laboratory animal feed (Beijing Keao Xieli Feed Co., Ltd., Beijing, China) at 22–26 ℃ with good ventilation and 40–70% humidity. The male and female mice were fed in separate cages, with free access to food and water. The padding was replaced regularly. The experiment was conducted after acclimatization for 1 week. The animal experimental processes were approved by the Ethics Committee of Southern Medical University and conducted in strict accordance with the standard of the Guide for the Care and Use of Laboratory Animals published by the Ministry of Science and Technology of the People’s Republic of China in 2006.

After being fed the high-fat and high-sugar diet for 4 weeks, mice were then mated at a ratio of 2:1. Female mice were observed the next morning, and those with pessary or sperm on vaginal secretion smear were considered successful mating. The first day was considered day 0 of pregnancy, the pregnant mice were marked, and the pregnancy period was recorded. All mice were given free access to food and water. The chow diet was full nutrition feed, and the high-fat and high-sugar diets were added with lard, egg yolk, and white sugar based on the common feed. Specifically, the self-made high-fat and high-sugar diet included 15% lard, 10% egg yolk, 10% white sugar, and 65% common experimental animal feed. After successful pregnancy, mice were continuously intraperitoneally injected with 30 mg/kg of freshly prepared streptozotocin (STZ) solution (Yeasen Company, Shanghai, China) once a day for 3 days. After 72 h of the last injection of STZ solution, blood glucose levels in the mice were measured using a glucometer. Successful model creation was determined when random blood glucose levels were ≥ 5.1 mmol/l.

### Grouping of experimental animals for oral administration of probiotics

The experimental animals were divided into three groups for oral probiotics administration. Each group consisted of 10 mice: control pregnancy group (normal pregnant mice), gestational diabetes mellitus (GDM) group (GDM mice), and probiotics group (GDM mice receiving daily oral administration of probiotics in their food). The probiotics used in this study were common strains of Bifidobacterium FTJS7K1 and FTJS5K1, extracted and purified from human feces. The mice in the probiotics group were orally administered the bacterial solution once a day at 1 × 109 CFU/mL (10 mL/kg) for 7 consecutive days (Wang et al. 2022a, b, c, d, e). After 7 days of model establishment, fresh fecal samples were collected from each group of mice. The ATB semi-automatic microbial detection system (Merieux, France) was used to measure Bifidobacterium, Escherichia coli, and Lactobacillus levels. Samples from different body parts of the mice in each group were collected for further experiments.

### Pregnancy outcomes, fetal body weight, and placental quality

Following the same experimental grouping as before, on the 20th day of pregnancy, the pregnant mice were subjected to cesarean section. The placentas were detached, and all fetuses were extracted. The survival of the fetuses was determined by observing whether they exhibited signs of breathing or voluntary movement, and the number of stillbirths was recorded. Fetal body weight was measured per litter, and placental weight was also measured.

### Glycolipid metabolism and insulin resistance measurement

The indicators for glucose and lipid metabolism and insulin resistance were assessed. In the experimental groups, 3 mL of venous blood was extracted, and the serum was obtained after centrifugation at 4 ℃ for 15 min at a speed of 3000 r/min and a centrifugal radius of 15 cm. The obtained supernatant was then subjected to machine detection. The Japanese Olympus AU5821 fully automated biochemical analyzer was used to measure total cholesterol (TC), triglycerides (TG), and low-density lipoprotein cholesterol (LDL-C). The Zhengzhou Antu A2000 fully automated chemiluminescence analyzer, along with the corresponding reagents, was used to measure fasting insulin (FINS), fasting blood glucose (FBG), and 2-h postprandial blood glucose (2hPG). The Japanese Aikola HA-8180 fully automated glycated hemoglobin analyzer was used to measure HbA1c. The homeostasis model assessment-insulin resistance (HOMA-IR) index was calculated as (FBG × FINS) / 22.5 (Li et al. 2021).

### Sample acquisition and transcriptome sequencing

Intestinal tissue samples from GDM and normal mice (n = 3) were collected. The total RNA was isolated using the Trizol reagent (Invitrogen, Carlsbad, CA). The RNA sample concentration was determined by an OD260/280 using a Nanodrop ND-1000 spectrophotometer (Thermo Fisher Scientific, Waltham, MA). RNA concentrations were determined using the Qubit RNA assay kit. Total RNA samples that meet the following requirements were used for subsequent experiments: RNA Integrity Index (RIN) ≥ 7.0 and 28 S: 18 S ≥ 1.5. CapitalBio Technology (Beijing, China) generated and sequenced the sequencing libraries. A total of 5 µg RNA was used per sample. Briefly, Ribo-Zero™ magnetic kit (Epicentre Technologies, Madison, Wisconsin) was used to remove ribosomal RNA from the total RNA. The sequencing library was constructed using Illumina’s NEB Next Ultra RNA Library Preparation Kit (NEB). Next, RNA fragments were converted into fragments with a length of about 300 base pairs (bp) in NEB. Next, the first chain synthesis reaction buffer (5 x). The first strand of cDNA was synthesized using reverse transcriptase primer and random primer, and the second strand of cDNA was synthesized in the second strand synthesis reaction buffer of dUTP Mix (10 x). End repair of cDNA fragments, including adding ployA tail and ligating sequencing adaptors. After joining the Illumina sequencing connector, the second strand of the cDNA was digested using USER Enzyme (NEB) to construct a strand-specific library. Library DNA was amplified, and PCR purified and enriched the libraries. Libraries were then identified by Agilent 2100 and quantified using the KAPA Library Quantitative Kit (KAPA Biosystems, South Africa). Finally, paired-end sequencing was performed on an Illumina NextSeqCN500 sequencer.

### Data analysis of transcriptome sequencing

The quality of the paired-end reads of the raw sequencing data was checked using the FastQC software v0.11.8 (www.bioinformatics.babraham.ac.uk). The raw data was processed using the Cutadapt software 1.18 (www.bioinformatics.babraham.ac.uk): removal of the Illumina sequencing connector and the poly (A) tail sequences. A perl script removed the reads with over 5% N content. The reads with 70% base mass above 20 were extracted using the FASTX Toolkit software 0.0.13 (http://hannonlab.cshl.edu/fastx_toolkit/). Double-end sequences were repaired using the BBMap software. Finally, the filtered high-quality reads fragments were aligned to the reference genome by the hisat2 software (0.7.12).

The mRNA-based read counts number was used for the differential expression analysis of the mRNA using the R language “edgeR” package (http://www.bioconductor.org/packages/release/bioc/html/edgeR.html), with the settings |log_2_FC| > 1 and *P*.value < 0.05 as the differential gene screening criteria. The KEGG pathway enrichment analysis of the differentially expressed genes (DEGs) was performed using the “ClusterProfiler” package (https://bioconductor.org/packages/release/bioc/html/clusterProfiler.html) in the R software, and *p* < 0.05 was considered statistically significant.

### Flow cytometric sorting for regulatory immune cells

The cardiac blood was collected from each group. The lymphocytes were isolated from the blood using gradient centrifugation. After being washed with PBS three times, lymphocytes were divided into three parts: one labeled with antibodies to CD4-PE (MA5-17450), CD25-APC (17-0251-82), and gFoxp3-FITC (71-5775-40); one labeled with antibodies to CD4-PE, CXCR5-APC(17-7185-82), Foxp3-FITC; one labeled with antibodies to CD19-PE (MA5-17794) and IL-10-APC (17-7101-81) (Thermo Fisher Scientific and Glory Science Co., Ltd). They were incubated at room temperature for 15 min. The proportion of CD4^+^CD25^+^Foxp^3+^Treg cells, CD4^+^CXCR5^+^Foxp^3+^Tfr cells, and CD19^+^IL-10^+^Breg in the lymphocytes was analyzed in a flow cytometer.

### Enzyme-linked immunosorbent assay (ELISA)

Serum levels of IL-6, TNF-α, IL-10, TGF-β, and leptin in mice were measured according to the manufacturer’s instructions based on mouse ELISA kits (E-EL-M2453c, E-EL-M3063, E-MSEL-M0031, E-EL-M3008; Elabscience, Wuhan, China). The optical density (OD) value was measured at the wavelength of 450 nm.

### Statistical analysis

Statistical analysis was performed using the R 3.6.0 Statistical Package (The R Foundation for Statistical Computing, Vienna, Austria) and SPSS 21.0 software (IBM Corp. Armonk, NY). Measurement data were expressed as mean ± standard deviation. Data were tested for normality using a Kolmogorov-Smirnov test and homogeneity of variance using F-test. The data conforming to normal distribution and homogeneity of variance between the two groups were compared using the unpaired *t*-test. A one-way analysis of variance (ANOVA) followed by Tukey’s post hoc test was performed for multiple comparisons. The results were considered statistically significant when *p* < 0.05.

## Results

### Significant differences in gut microbiota composition between GDM patients and healthy individuals

**Fig. 2:**
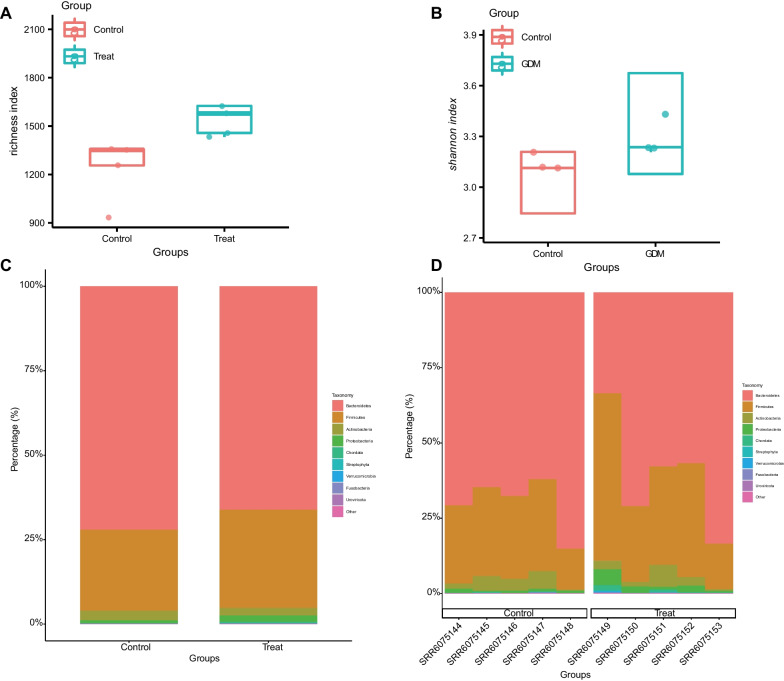
Comparison of species diversity of gut microbiota between GDM patients and healthy individuals. **A** Alpha diversity analysis of richness index in the GDM patients (n = 5) and healthy individuals (n = 5). **B** Alpha diversity analysis of Shannon index in the GDM patients (n = 5) and healthy individuals (n = 5). **C** The species abundance stacked plot at the “phylum” level with the grouping as the horizontal axis. Different colors represent the gut microbiota of different phyla. **D** The species abundance stacked plot at the “phylum” level with the sample as the horizontal axis. Different colors represent the gut microbiota of different phyla

To assess differences in microbial species diversity between GDM patients and healthy individuals, sequences were aligned to estimate alpha diversity. Alpha diversity calculates the species composition within samples, including two-dimensional information on number and abundance (Parikh et al. 2020). The alpha diversity analysis showed an evident difference between the two groups richness and Shannon indexes (Fig. 2A, B), which indicated that the number and abundance of gut microbiota species varied significantly between GDM patients and healthy individuals.

In addition, the species composition analysis found that in the species abundance stacked plot at the “phylum” level with the group as the horizontal axis, there was evident difference in gut microbiota species composition at the phylum level between GDM patients and healthy individuals (Fig. 2C), *Fusobacteria* and *Firmicutes* were more abundant in GDM patients. At the same time, Bacteroidetes were more abundant in healthy individuals. Meanwhile, analysis of the species abundance stacked plot of samples showed no difference in the gut microbiota composition within groups. Still, there were obvious differences in gut microbiota composition between groups (Fig. 2D). Thus, there were evident differences in gut microbiota composition between GDM patients and healthy individuals.

### Significant differences in species abundance of gut microbiota between GDM patients and healthy individuals

**Fig. 3:**
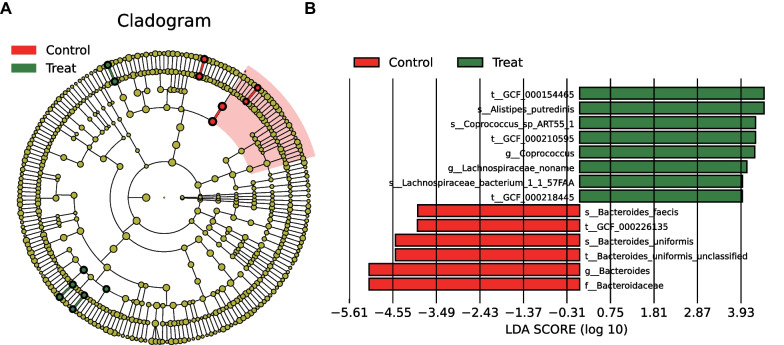
Differences in species abundance of gut microbiota between GDM patients and healthy individuals. **A** Branch diagram of species abundance classification of gut microbiota in GDM patients (n = 5) and healthy individuals (n = 5). The circle radiating inside to outer represents the classification level from phyla to genus, and the diameter represents the relative abundance. Yellow nodes indicate species without significant differences, red nodes indicate microbiota with higher abundance in healthy individuals, and green nodes indicate higher abundance in GDM patients. **B** LDA value distribution histogram of species abundance of GDM patients (n = 5) and healthy individuals (n = 5)

LEfSe analysis was performed and visualized to explore the abundance difference of specific species. It was noted (Fig. 3A, B) that the relative abundance of *Bacteroides_caccae*, *Bacteroides_coprocola*, *Bacteroides_coprophilus*, *Bacteroides_plebeius*, *Bacteroides_stercoris*, *Megamonas_hypermegale*, *Bacteroides_cellulosilyticus* in the fecal samples of GDM patients was lower than that in the fecal samples of from healthy individuals (LDA score [log 10] > 2). The red part indicated healthy individuals and the green part indicated GDM patients. Whereas, *Prevotella_copri*, *Alistipes_putredinis*, *Alistipes_putredinis*, *Coprococcus_sp_ART55_1*, and *Dialister_invisus* were more abundant in the fecal samples of GDM patients (LDA score [log 10] > 2). The red part indicated healthy individuals and the green part indicated GDM patients.

These findings suggested obvious differences in the abundance of gut microbiota species between GDM patients and healthy individuals. These differentially abundant microbiota were sufficient to distinguish the microbiota of healthy individuals and GDM patients.

Moreover, existing evidence pointed out that the selected differentially abundant floras were significantly associated with fat accumulation and inflammatory response. It has been documented that *Bacteroides_cellulosilyticus* plays an important role in the degradation of polysaccharides such as cellulose (Robert et al. 2007), while *Prevotella_copri* is significantly associated with fat accumulation (Maldonado-Contreras et al. 2020). The high copri abundance is associated with increased concentrations of obesity-related serum metabolites (lipopolysaccharides, branched-chain amino acids, aromatic amino acids, and arachidonic acid metabolites) (Newman et al. 2021). The increases in intestinal barrier permeability and host chronic inflammation result in fat accumulation and serum metabolite alterations (Chen et al. 2021a, b).

### Significant differences in the functional composition of the microbiota in GDM patients

**Fig. 4:**
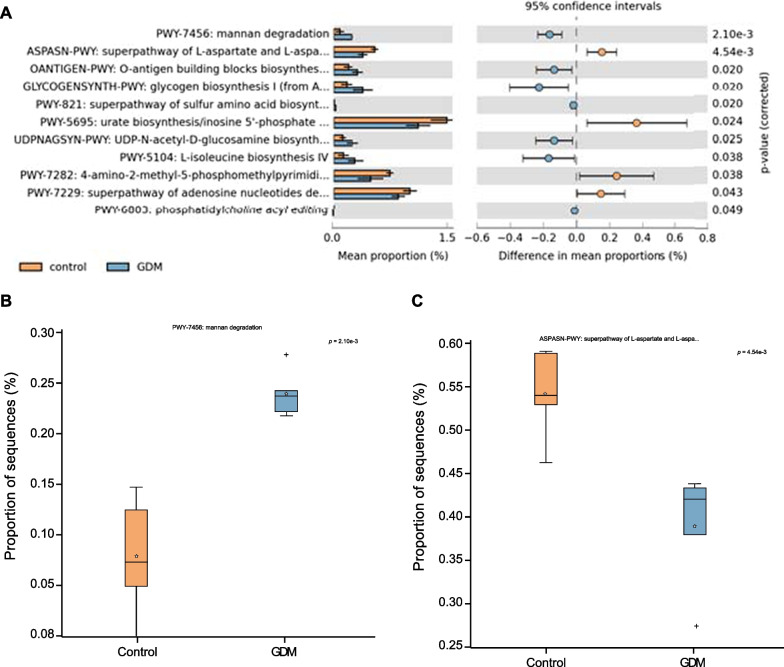
Functional analysis of gut microbiota in GDM patients and healthy individuals. **A** Functional analysis of gut microbiota in GDM patients (n = 5) and healthy individuals (n = 5). **B** The difference in mannan degradation between the samples from GDM patients (n = 5) and healthy individuals (n = 5). **C** Differences in the super pathway of L-aspartate and L-asparagine biosynthesis between the samples from GDM patients (n = 5) and healthy individuals (n = 5). *p* < 0.05

The investigation moved to the differences in the functional composition of the gut microbiota in GDM patients based on further visualization by STAMP software. As shown in Fig. 4A, we found that these differentially abundant gut microbiota were enriched in multiple functional pathways, among which mannan degradation and superpathway of L-aspartate and L-asparagine biosynthesis were ranked in the front position (*P* value). Thus, they were investigated as candidate routes (Homayouni et al. 2020). It was previously found that mannan degradation was closely related to GDM and was more enriched in GDM patients, which is consistent with our analysis results (Fig. 4B). Meanwhile, a recent study has confirmed that the super pathway of L-aspartate and L-asparagine biosynthesis pathway were less enriched in GDM patients (Lautrup et al. 2019), which was also consistent with our analysis results (Fig. 4C). The above results indicate that differentially abundant gut microbiota may participate in the development of GDM through mannan degradation, super pathway of L-aspartate, and L-asparagine biosynthesis.

### Dysregulation of gut microbiota may promote the development of GDM by upregulating leptin

**Fig. 5:**
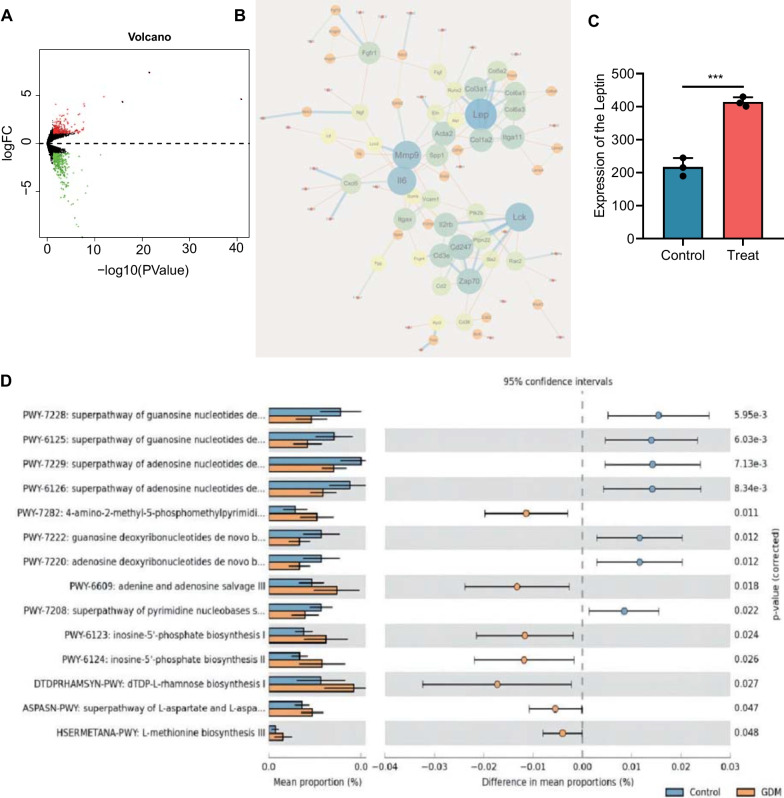
Differential gene analysis of transcriptomic sequencing in GDM and healthy individuals. **A** Volcano map of the DEGs in GDM mice (n = 3) and normal control mice (n = 3) by transcriptomic sequencing (Blue represents the downregulated genes, and red represents the upregulated genes). **B** PPI network of the proteins encoded by the DEGs in GDM mice (n = 3) and normal control mice (n = 3) through transcriptome sequencing analysis (The size represents the degree value, and the color from deep to shallow represents combined_score from low to high). **C** Leptin protein level in the peripheral blood of GDM mice (n = 3) and normal control mice (n = 3) measured by ELISA (*** *p* < 0.05). **D** Functional analysis of the gut microbiota of GDM mice (n = 3) and normal control mice (n = 3)

To further explore the possible molecular mechanisms by which gut microbiota dysbiosis regulates the development of GDM, GDM mouse models were established. The GDM mouse models with random blood glucose higher than 5.1 mmol/L were established successfully. The mRNA expression in GDM and healthy individuals was analyzed by whole transcriptome resequencing. There were 573 DEGs (including 346 downregulated DEGs and 227 upregulated DEGs) (Fig. 5A). The results of the PPI network analysis exhibited that F2, Edn1, Lgf1, Wnt2, Eln, and leptin occupied significant positions (Fig. 5B), among which leptin was in the middle (combined_score was the largest). Meanwhile, the leptin expression was measured using ELISA, which showed that the protein level of leptin was significantly increased in GDM mice (Fig. 5C), consistent with the sequencing results.

It has been reported that leptin and its receptor proteins are closely associated with the L-aspartate and L-asparagine biosynthesis pathway’s super pathway (Trusov et al. 2021). In addition, fecal samples were collected from GDM patients and healthy individuals for metagenomic sequencing. Enrichment analysis of the gut microbiota revealed that the selected gut microbiota was mainly enriched in C4 photosynthetic carbon assimilation cycle and adenine and adenosine salvage III. The mannan degradation, the super pathway of L-aspartate and L-asparagine biosynthesis functional pathways that we were focused on in the patient samples also showed consistent enrichment results (Fig. 5D). There is a similar dysbiosis of gut microbiota in GDM patients, and GDM mice and an increased protein level of leptin have been found in GDM mice. Therefore, we hypothesize that dysbiosis of gut microbiota may lead to upregulation of Leptin expression, impacting the occurrence and development of GDM.

### Oral probiotics repair gut microbiota dysbiosis and inhibit the inflammatory response to maintain normal pregnancy in GDM mice

Evidence demonstrates that the gut microbiota is severely disturbed in GDM patients, activating the body’s inflammatory response and further damaging the pancreatic islet cells to exacerbate the progression of diabetes (Taylor et al. 2017). As an independent factor that can effectively alleviate insulin resistance and regulate intestinal microecology, probiotics are a new idea to prevent GDM (Kijmanawat et al. 2019). Therefore, GDM mice induced by high-fat and high-sugar diets were fed with probiotics to observe the effects of probiotics on GDM mice.

First, the composition of gut microbiota (according to the above conclusions, *Bacteroides_caccae*, *Prevotella_copri*, and *Bacteroides_cellulosilyticus* were significantly different between the normal mice and GDM mice and belonged to the common gut microbiota in the organism, which has reported to be related to GDM (McKay et al. 2017; Sedighi et al. 2017). The contents of *Bacteroides_caccae*, *Prevotella_copri*, and *Bacteroides_cellulosilyticus* (LgCFU, the logarithm of colony formation units per gram of feces of wet weight) in the fecal samples of control pregnant mice were 10.350 ± 0.8374, 7.900 ± 0.4351, and 7.106 ± 0.4524, while those of GDM mice were 5.600 ± 0.9223, 10.050 ± 0.7693, 4.750 ± 0.6212. The difference between the two groups was statistically significant. The contents of *Bacteroides_caccae*, *Prevotella_copri*, and *Bacteroides_cellulosilyticus* in the fecal samples of GDM mice treated with probiotics were 12.770 ± 1.7460, 6.667 ± 0.6912, and 7.367 ± 0.3739. Compared with the GDM mice, the difference was statistically significant, while the control pregnant mice were not statistically significant (Additional file 1: Table S1). It indicated that the gut microbiota composition of GDM mice has been improved again by supplementing probiotics. *Bacteroides_caccae* and *Bacteroides_cellulosilyticus* increased the content by supplementing probiotics to reach the standard of normal mice, while *Prevotella_copri* reduced the content to reach the standard of normal mice by supplementing probiotics.

After testing the pregnancy outcomes of mice, the results are shown in Additional file 1: Table S2. The rate of viable fetuses in the GDM group was significantly lower than in the control and probiotic groups, with statistical significance (P < 0.05). However, there was no statistically significant difference in the rate between the control and probiotic groups (P > 0.05). The fetal mouse body weight and placental weight in the GDM group were significantly higher than in the control and probiotic groups, with statistical significance (P < 0.05). Again, there was no statistically significant weight difference between the control and probiotic groups (P > 0.05). These findings indicate that supplementing with probiotics improved the pregnancy outcomes of mice and maintained pregnancy stability. Additionally, when examining markers of glucose and lipid metabolism and insulin resistance, as shown in Additional file 1: Table S3, the control group and the probiotic group showed lower TC, TG, LDL-C, FBG, 2hPG, HbA1c, FINS, and HOMA-IR index values compared to the GDM group, with statistical significance (P < 0.05). However, there was no statistically significant difference in these markers between the control and probiotic groups (P > 0.05). This result suggests that supplementing with probiotics alleviated the disease in GDM mice.

It should be noted that Treg cells, Tfr cells, and Breg cells regulate and cooperate to constitute a regulatory immune cell network, maintaining the homeostasis of the immune system and the timely termination of the immune response (Lee et al. 2022). The Treg cells can promote the proliferation of the Tfr cells, and both the Tfr cells and the Breg cells jointly regulate the follicular helper T cells and the B cells to maintain the homeostasis of humoral immunity (Li et al. 2021; Liao and Tsai 2023; Liu et al. 2021). In GDM patients, the number and impaired function of regulatory immune cells such as Treg cells is reduced, causing abnormal inflammatory reactions in pregnant women and various fetal sequelae (Luo et al. 2021).

**Fig. 6:**
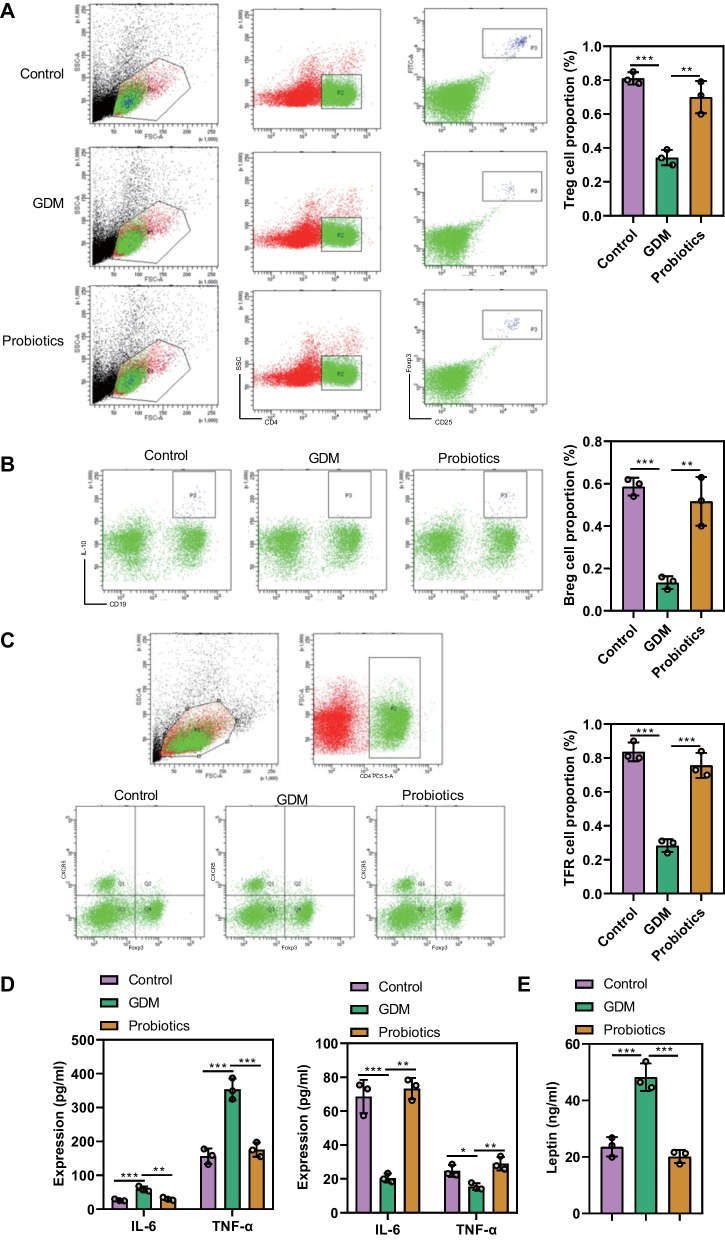
Quantitative analysis for immune cells and immune molecules implicated in GDM mice in response to oral probiotics. **A** Treg immune cells in control pregnant mice, untreated GDM mice, and GDM mice treated with oral probiotics were detected by flow cytometry. **B** Breg immune cells in control pregnant mice, untreated GDM mice, and GDM mice treated with oral probiotics detected by flow cytometry. **C** Tfr immune cells in control pregnant mice, untreated GDM mice, and GDM mice treated with oral probiotics detected by flow cytometry. **D** The concentrations (ng/mL) of IL-6, TNF-α, IL-10, and TGF-β in the serum of control pregnant mice, untreated GDM mice, and GDM mice treated with oral probiotics measured by ELISA. **E** Leptin protein levels in control pregnant mice, untreated GDM mice, and GDM mice treated with oral probiotics measured by ELISA. * *p* < 0.05, ** *p* < 0.01, *** *p* < 0.001. n = 3

Next, we aimed to study the effect of oral probiotics on regulatory immune cells. Flow cytometric data displayed that the proportion of CD4^+^CD25^+^Foxp^3+^Treg cells in lymphocytes of control pregnant mice, GDM mice, and GDM mice treated with probiotics was (0.81 ± 0.04)%, (0.34 ± 0.05)% (*p* = 0.0003, comparison between control pregnant mice and GDM mice), (0.70 ± 0.10)% (*p* = 0.0012, comparison between GDM mice and GDM mice treated with probiotics), respectively (Fig. 6A). Moreover, the proportion of CD19^+^IL-10^+^Breg cells in lymphocytes of control pregnant mice, GDM mice, and GDM mice treated with probiotics was (0.59 ± 0.04)%, (0.13 ± 0.03)% (*p* = 0.0006, comparison between control pregnant mice and GDM mice), and (0.52 ± 0.12)% (*p = *0.0016, comparison between GDM mice and GDM mice treated with probiotics), respectively (Fig. 6B). The proportion of CD4^+^CXCR5^+^Foxp^3+^Tfr cells in lymphocytes of control pregnant mice, GDM mice, and GDM mice treated with probiotics was (0.84 ± 0.06)%, (0.28 ± 0.04)% (*p* < 0.0001, comparison between control pregnant mice and GDM mice), and (0.76 ± 0.07)% (*p* = 0.0001, comparison between GDM mice and GDM mice treated with probiotics), respectively (Fig. 6C). It suggested that oral probiotics can promote the increase of the proportion of Treg cells, Tfr cells and Breg cells in GDM mice, so as to maintain the homeostasis of humoral immunity and the normal pregnancy of mice.

In addition, ELISA presented that the concentrations of IL-6, TNF-α, IL-10, and TGF-β in the serum of control pregnant mice were26.85 ± 3.03 (pg/mL), 156.28 ± 22.74 (pg/mL), 68.59 ± 9.86 (pg/mL) and 24.67 ± 3.47 (pg/mL). Those in the serum of GDM mice were 59.37 ± 7.62 (pg/mL), 353.69 ± 31.74 (pg/mL), 20.48 ± 2.61 (pg/mL), and 15.36 ± 2.01 (pg/mL) (*p* < 0.05). In contrast, those in the serum of GDM mice treated with probiotics were 30.42 ± 4.29 (pg/mL), 175.81 ± 21.14 (pg/mL), 73.26 ± 6.28 (pg/mL) and 28.95 ± 3.96 (pg/mL) (*p*=0.0014, *p* = 0.0004, *p* = 0.0002, *p* = 0.0052) (Fig. 6D). It revealed that oral probiotics could reduce the levels of inflammatory molecules, such as TNF-α and IL-6, but elevate the levels of immunosuppressive molecules, such as TGF-β and IL-10, which can help to regulate the body’s immune response and maintain normal pregnancy in mice.

ELISA also exhibited that the leptin protein content (ng/mL) of control pregnant mice and GDM mice was 23.59 ± 3.43 and 48.27 ± 4.88, respectively (*p* < 0.05). The leptin protein content (ng/mL) of GDM mice treated with probiotics was 20.12 ± 2.31 (*p* < 0.05) (Fig. 6E), indicating that oral probiotics could reduce leptin protein to relieve GDM.

It can be concluded that oral probiotics could promote the increased proportion of Treg cells, Tfr cells, and Breg cells in GDM mice and reduce the inflammation-related molecules and leptin protein content, which can inhibit the inflammatory response and maintain normal pregnancy in mice.

## Discussion

The study of gut microbiota composition, species abundance, and functional composition is essential to understand the development of GDM in individuals (Mokkala et al. 2021). Giannella et al. summarized the alterations in the pregnancy microbiota based on their study. They proposed that it is crucial to regulate the microbiota to prevent and treat diseases, including GDM. This regulation would contribute to the development of personalized therapies (Giannella et al. 2023). This study found that the gut microbiota is significantly different in GDM patients and healthy individuals, with higher levels of chondrocytes and ligamentous bacteria in GDM patients and higher levels of Bacteroidetes in healthy individuals (Tang et al. 2020). Significant variations exist in gut microbiota composition between pregnant women affected by gestational diabetes mellitus (GDM) and those without the condition (Vavreckova et al. 2022). Dysbiosis of the gut microbiota may affect the development of GDM by altering the metabolic pathways of L-aspartate and L-aspartate biosynthesis (Han et al. 2020). Wang’s findings also revealed that acetic acid, propionic acid, and butyric acid in the circulation of women with GDM influence placental immune metabolism, exhibiting potential anti-diabetic and anti-inflammatory properties (Wang et al. 2022a, b, c, d, e). In addition, this study found that oral probiotics reduced leptin levels and prevented GDM by increasing the ratio of Treg, Tfr, and Breg cells, suppressing the inflammatory response, and maintaining normal pregnancy in mice (Kanda et al. 2021).

Similarly, research has shown that postpartum gut dysbiosis still exists and may affect the development of newborns (Farhat et al. 2022). In the evolution process of GDM, probiotic supplements are appropriate for blood sugar control and provide the most powerful evidence for fetal development and postpartum (Mu et al. 2023). Understanding these mechanisms and the role of gut microbiota in GDM can help develop preventive measures and treatments for GDM (Mustad et al. 2020). Therefore, studying the gut microbiota and its impact on GDM is essential to improve the health outcomes of mothers and children (Graham et al. 2021). Metagenomic and transcriptomic sequencing is emerging as a promising tool to assess the contribution of the gut microbiota to disease (Frostegard et al. 2022).

There is growing evidence that the gut microbiota can influence host glucose metabolism and that dysregulation of the gut microbiota is associated with the pathogenesis of GDM (Homayouni et al. 2020; Wang et al. 2020; Mora-Janiszewska et al. 2022). An imbalanced gut microbiota is thought to affect the movement of lipopolysaccharide (LPS) through the gut wall, leading to mucosal inflammation and endotoxemia (Morelli et al. 2019). Based on metagenomic and transcriptomic sequencing and in vivo experiments, oral probiotics were found to inhibit the inflammatory response to suppress leptin protein in GDM, thereby maintaining normal pregnancy in mice (Perez-Perez et al. 2020). According to research, women with impaired glucose tolerance (IGT) may temporarily avoid a diagnosis of gestational diabetes mellitus (GDM) by modifying their gut microbiota (Dreisbach et al. 2022). Consequently, the oral glucose tolerance test (OGTT) was omitted for the GDM mice in this study. In this study, we did not measure L-glutamate levels and related metabolites. Instead, we analyzed the differences in gut microbiota functionality in GDM patients and found that the L-glutamate biosynthesis pathway was significantly enriched. It allowed us to further explore the relationship between leptin and its receptor proteins with GDM. Additionally, the L-glutamate data obtained from database mining aligned with the transcriptomic data from our mouse model, further validating our hypothesis.

Alpha-diversity and beta-diversity showed significant differences in the composition, species abundance, and functional composition of the gut microbiota between GDM patients and healthy individuals (Mustad et al. 2020). Notably, gut microbiota dysbiosis in women with GDM is mainly characterized by changes in microbiota diversity, including alpha-diversity, species diversity within the same individual, and beta-diversity, species diversity between individuals (Crusell et al. 2020). Gambardella et al. provided relevant answers to technical issues about alpha and beta diversity of the gut microbiota and standardization of research outcomes (Gambardella et al. 2021). Disturbances in the normal gut microbiota composition may lead to host metabolic dysregulation, mainly responsible for various diseases, including GDM (Medici Dualib et al. 2021). A growing number of studies suggest that dysregulated gut microbiota increases adiposity, β-cell dysfunction, metabolic endotoxemia, systemic inflammation, and oxidative stress, ultimately driving the development of T2DM (Galicia-Garcia et al. 2020; Fang et al. 2022; Zhou et al. 2022). Furthermore, the question of whether gestational diabetes mellitus (GDM) can result in neonatal diabetes mellitus (NDM) or postpartum diabetes is of significant importance (Bukhari et al. 2022a, b). The higher relative abundance of Prevotellaceae was associated with obesity and impaired glucose metabolism (Zheng et al. 2021). Significant differences in Bacteroides_caccae, Prevotella_copri, and Bacteroides_cellulosilyticus between healthy individuals and GDM patients have been reported to be associated with GDM (Sweeting et al. 2022). A previous study of a sample of Mexican women found a high abundance of Fusobacterium in the pregnancy health condition, followed by Eubacterium and Bacteroides (Benitez-Guerrero et al. 2022). Multi-strain probiotics can regulate gut dysbiosis and improve metabolic and inflammatory outcomes in women with gestational diabetes mellitus (GDM) (Hasain et al. 2022). In addition to modulating the gut microbial community and diversity, additional oral probiotic treatment may alleviate symptoms of metabolic disorders associated with T2DM (Zhou et al. 2022).

Furthermore, the data obtained verified that the gut microbiota influences the development of GDM by altering the metabolic pathways of the super pathway of L-aspartate and L-aspartate biosynthesis (Yang et al. 2022). The balance of asparagine and aspartate is associated with T2DM, while the role of the super pathway of L-aspartate and L-aspartate in GDM remains further elucidated (Rhee et al. 2021). However, our study primarily obtained fecal sample data from GDM patients and normal healthy controls through the SRA database. We did not conduct a comprehensive exploration of the intestinal microbiota in GDM patients, potentially neglecting some potential pathways related to GDM. In addition, transcriptome sequencing data also verified that leptin is upregulated in GDM (Luo et al. 2021). Leptin is a hormone that controls satiety and is secreted mainly by adipocytes in response to adequate energy stores to reduce appetite through hypothalamic stimulation of anorexigenic peptides (Wang et al. 2022a, b, c, d, e). There is evidence of an association between dysregulated gut microbiota and leptin expression under inflammatory conditions (Heiss et al. 2021). A recent study reported that leptin levels are elevated during pregnancy, which can exacerbate pregnancy-related insulin resistance and the onset of GDM, consistent with our findings (Pan et al. 2021).

Further, in vivo experiments verified that oral probiotics could inhibit leptin expression and hinder the development of GDM (Wieers et al. 2019). Meanwhile, oral probiotics could increase the Treg, Tfr, and Breg cell ratio in GDM mice, inhibit the inflammatory response, and maintain normal pregnancy in mice (Trend et al. 2018). Oral administration of probiotics significantly reduced leptin receptor gene expression in mice with colon cancer (Ranji et al. 2019). In addition, leptin expression was significantly reduced in obese women with food addiction in the presence of probiotics (Narmaki et al. 2022). During the second half of pregnancy, increased levels of pro-inflammatory cytokines decreased maternal insulin sensitivity (Propper and Balkwill 2022). Decreased number of Treg cells in the placenta is associated with GDM (De Luccia et al. 2020). Probiotics are beneficial microorganisms with good bioactivity to prevent metabolic diseases (Hong et al. 2022). One study found that regular consumption of probiotics is beneficial in regulating intestinal microbiota composition (Wieers et al. 2019). Probiotics may positively affect metabolism, inflammation, oxidative stress, and neonatal outcomes in women with GDM (Sweeting et al. 2022). Maternal dietary interventions can potentially reduce gut dysbiosis, thus decreasing the risk of GDM and its associated complications for both the mother and the infant (Bankole et al. 2022).

Moreover, a high-complex carbohydrate and low-fat diet in women diagnosed with GDM contributes to a favorable microbial environment for metabolism, characterized by increased bacterial diversity and potential reduction in pathogenic organisms during the initial four months postpartum (Sugino et al. 2022). The study found that administering probiotic supplementation improved pregnancy outcomes and various glucose and lipid metabolism indicators, including insulin resistance. Wan performed an intervention involving 52 pregnant women, where galactooligosaccharides (GOS) were administered as a prebiotic supplement (Wan et al. 2023). However, no significant benefits on glucose and lipid metabolism were observed. Probiotics favorably induce Treg cells and elevate anti-inflammatory cytokines and growth factors, including IL-10 and TGF, in patients with GDM (Liao and Tsai 2023). The potential of probiotics in regulating the gut microbiota and modulating pro-inflammation has also been demonstrated in gestational diabetes (Wieers et al. 2019). Furthermore, there is evidence that probiotics could be a promising therapeutic tool to improve T2DM due to their ability to modulate the gut microbiota, produce short-chain fatty acids (SCFAs) and glucagon-like peptides, elevate SIRT1, inhibit alpha-glucosidase and fetuin-A levels, and downregulate pro-inflammatory cytokine levels (Bajinka et al. 2023). Therefore, it can be concluded that oral probiotics can alleviate gut microbiota dysbiosis and inflammatory response by down-regulating leptin protein expression, thus curbing the development of GDM (Lv et al. 2022).

During pregnancy, sex hormones regulate the interplay and coordination among various regulatory immune cells, such as Treg cells, Tfr cells, and Breg cells, forming a network of regulatory immune cells that maintain immune system homeostasis and timely termination of immune responses. Treg cells promote the proliferation of Tfr cells, while Tfr cells and Breg cells jointly regulate follicular helper T cells and B cells, thereby maintaining the stability of humoral immunity. In women with gestational diabetes mellitus (GDM), there is dysbiosis of intestinal microbiota, leading to a reduction in the number and impaired function of regulatory immune cells, including Treg cells, resulting in abnormal maternal inflammatory responses and various fetal sequelae (Schober et al. 2014; Paolino et al. 2021). Therefore, we chose to investigate the impact of oral probiotics on regulatory immune cells in Treg, Tfr, and Breg cells to elucidate the connection between dysbiosis of intestinal microbiota and regulatory immune cells, such as Treg cells.

## Conclusion

**Fig. 7:**
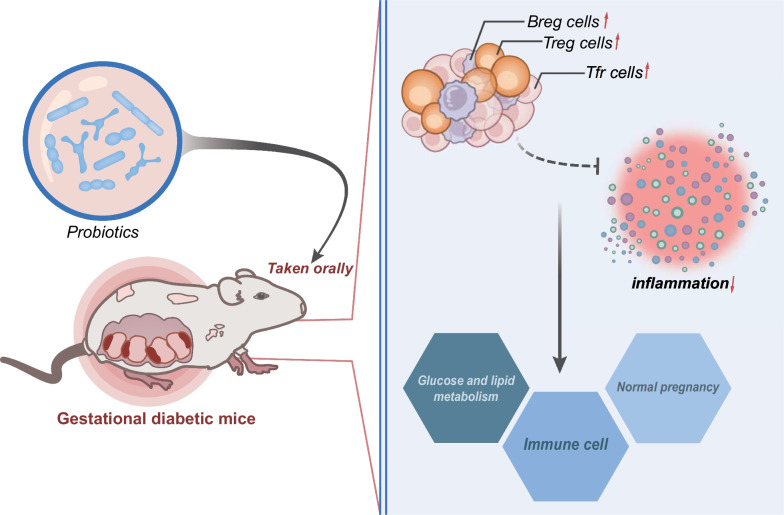
Schematic representation of the potential mechanisms involved in the effect of oral probiotics in GDM. Gut microbiota dysbiosis leads to increased leptin expression and participates in the development of GDM. Oral probiotics increase the proportion of Treg, Tfr, and Breg cells in GDM mice to inhibit inflammation, thus maintaining normal pregnancy in mice

In summary, our study shows that dysregulation of the gut microbiota leads to increased leptin expression, which is involved in the development and progression of GDM. However, oral administration of probiotics can increase the ratio of Treg cells, Tfr cells, and Breg cells in GDM mice and suppress the inflammatory response, thus maintaining normal pregnancy and alleviating GDM in mice (Fig. 7). This study reveals potential molecular mechanisms underlying gestational diabetes mellitus (GDM), including changes in the gut microbiota, abundance and composition of intestinal microbiota, and overexpression of leptin protein, which may affect the development of GDM. These findings contribute to our understanding of GDM, particularly its impact on maternal and fetal health. Our study also suggests that oral probiotics may help prevent the development of GDM, possibly by increasing the proportion of regulatory immune cells and suppressing inflammatory responses. This study offers a new and promising strategy for preventing GDM and improving the health of both mothers and fetuses.

However, there are limitations to this study. Firstly, although the results are derived from experiments in a mouse model, the physiological mechanisms in mice may not fully replicate those in humans. Thus, the findings may not be directly applicable to humans. Further validation in human samples is needed in the future. Additionally, while the study found significant differences in the gut microbiota between GDM patients and healthy individuals, it does not determine whether these differences cause GDM or result from GDM. Therefore, further validation in humans is necessary to establish the exact relationship between changes in gut microbiota and GDM, as well as the effectiveness of oral probiotics in preventing the development of GDM. If positive results are obtained in human studies, clinical trials can be designed to test the efficacy and safety of oral probiotics for GDM prevention. If successful, oral probiotics can be a new intervention for preventing GDM.

### Supplementary information

**Additional file 1: Table S1.** Quantitative analysis of gut microbiota structure in pregnant mice. **Table S2.** Quantitative analysis of live birth rate, fetal body weight, and placental weight of pregnant mice. **Table S3.** Quantitative analysis of glycolipid metabolism and insulin resistance in pregnant mice.

## Data Availability

The data supporting this study’s findings are available on request from the corresponding author.
