# Supplementary material for: Oral probiotics increased the proportion of Treg, Tfr, and Breg cells to inhibit the inflammatory response and impede gestational diabetes mellitus
Source: Mol Med. 2023 Sep 8;29:122. doi: 10.1186/s10020-023-00716-4 (PMC10492300; doi:10.1186/s10020-023-00716-4)
Supplement: Supplementary file 1 — Additional file 1: Table S1. Quantitative analysis of gut microbiota structure in pregnant mice. Table S2. Quantitative analysis of live birth rate, fetal body weight, and placental weight of pregnant mice. Table S3. Quantitative analysis of glycolipid metabolism and insulin resistance in pregnant mice. [file 10020_2023_716_MOESM1_ESM.docx]

**Table S1** Quantitative analysis of gut microbiota structure in pregnant mice

| Gut microbiota | Groups | | | *P* value | | |
| --- | --- | --- | --- | --- | --- | --- |
|  | Control group | GDM group | Probiotics group | Control group *vs.* GDM group | Control group *vs.* Probiotics group | GDM group *vs.* Probiotics group |
| *Bacteroides_caccae* | 8.262 ± 0.788 | 5.135 ± 0.747 | 9.862 ± 1.271 | <0.0001 | 0.4842 | <0.0001 |
| *Prevotella_copri* | 6.915 ± 0.914 | 11.348 ± 1.317 | 5.819 ± 0.495 | <0.0001 | 0.1340 | <0.0001 |
| *Bacteroides_cellulosilyticu* | 7.591 ± 1.030 | 4.261 ± 0.374 | 7.616 ± 0.848 | <0.0001 | 0.7940 | <0.0001 |

Note: *p* < 0.05 indicates significant difference; *p* > 0.05 indicates no significant difference.

**Table S2** Quantitative analysis of live birth rate, fetal body weight, and placental weight of pregnant mice

| Variables | Groups | | | *P* value | | |
| --- | --- | --- | --- | --- | --- | --- |
|  | Control group | GDM group | Probiotics group | Control group *vs.* GDM group | Control group *vs.* Probiotics group | GDM group *vs.* Probiotics group |
| Live birth rate (%) | 90.23 ± 8.36 | 76.53 ± 4.93 | 89.39 ± 9.52 | <0.0017 | 0.9718 | <0.0031 |
| Fetal body weight (g) | 1.340 ± 0.138 | 1.052 ± 0.093 | 1.291 ± 0.125 | 0.0001 | 0.6379 | 0.0004 |
| Placental weight (g) | 0.179 ± 0.021 | 0.310 ± 0.041 | 0.197 ± 0.020 | <0.0001 | 0.3548 | <0.0001 |

Note: *p* < 0.05 indicates significant difference; *p* > 0.05 indicates no significant difference.

**Table S3** Quantitative analysis of glycolipid metabolism and insulin resistance in pregnant mice.

| Variables | Groups | | | *P* value | | |
| --- | --- | --- | --- | --- | --- | --- |
|  | Control group | GDM group | Probiotics group | Control group *vs.* GDM group | Control group *vs.* Probiotics group | GDM group *vs.* Probiotics group |
| TC (mmol/L) | 1.710 ± 0.202 | 2.581 ± 0.220 | 1.800 ± 0.246 | <0.0001 | 0.6455 | <0.0001 |
| TG (mmol/L) | 0.910 ± 0.079 | 2.850 ± 0.407 | 1.211 ± 0.109 | <0.0001 | 0.1566 | <0.0001 |
| LDL-C (mmol/L) | 0.561 ± 0.055 | 1.718 ± 0.190 | 0.609 ± 0.076 | <0.0001 | 0.6592 | <0.0001 |
| FBG (mmol/L) | 6.811 ± 0.569 | 11.259 ± 1.371 | 7.110 ± 0.593 | <0.0001 | 0.7512 | <0.0001 |
| 2hPG (mmol/L) | 8.960 ± 0.953 | 18.242 ± 2.182 | 9.239 ± 1.306 | <0.0001 | 0.9167 | <0.0001 |
| HbA1c (%) | 10.629 ± 1.195 | 21.460 ±3.029 | 11.319 ± 0.996 | <0.0001 | 0.7153 | <0.0001 |
| FINS (mIU/L) | 1.215 ± 0.143 | 2.851 ± 0.404 | 1.350 ± 0.193 | <0.0001 | 0.5148 | <0.0001 |
| HOMA-IR | 2.149 ± 0.229 | 4.630 ± 0.561 | 2.289 ± 0.273 | <0.0001 | 0.6966 | <0.0001 |

Note: *p* < 0.05 indicates significant difference; *p* > 0.05 indicates no significant difference.
